# Urethral prolapse mimicking a uterine prolapse in an eight-year-old girl: A case report

**DOI:** 10.1016/j.ijscr.2022.107169

**Published:** 2022-05-06

**Authors:** John Kasereka Muteke, Albert Yemba Baruani Ahuka, Franck Katembo Sikakulya

**Affiliations:** a University of Goma, Faculty of Medicine, Department of Obstetrics and Gynecology, Democratic Republic of the Congo; b Regional Hospital of North-Kivu Province, Goma, Department of Obstetrics and gynecology, Democratic Republic of the Congo; c Université catholique du Graben, Faculty of Medicine, Department of Surgery, Democratic Republic of the Congo

**Keywords:** Urethral prolapse, Surgical management, Uterine prolapse, DR Congo

## Abstract

**Introduction and importance:**

A urethral prolapse is a rare condition occurring most likely in prepubertal girls and post-menopausal women. The cause of this condition is not well known but an under-laying low level of estrogen is thought to have a role.

**Case presentation:**

This is an 8-year-old girl diagnosed with urethral prolapse, who was successfully managed by excision of the prolapsed urethral mucosa circumferentially. A three-month following up did not notice any particular challenge.

**Clinical discussion:**

The most common presenting sign is genital bleeding and the vaginal doughnut sign. Treatment of urethral prolapse should begin with medical therapy in most patients before resorting to surgical management. However, in case of bigger size, severe genital hemorrhage, and prolapsed mucosa with an appearance suggestive of vascular compromise, surgical management is the first-line option. We estimate a severe prolapse mimicking a uterine prolapse must be included in surgical management as a first-line option.

**Conclusion:**

Surgical excision may be the first-line option in certain urethral prolapses given its association with quick recovery versus the effectiveness of the long-time required for estrogen use, as well as the low likelihood of successful resolution linked to estrogen use.

## Highlights

• Urethral prolapse is a rare condition.
• A low estrogen level is thought to have a role.
• Estrogen cream application is the treatment of choice.
• Surgical management may be a first-line option in severe conditions such as one mimicking a uterine prolapse.

## 1 Introduction

Urethral prolapse is a rare clinical condition presented as a round doughnut-shaped secondary to complete distal urethral mucosa protrusion through the external meatus of the urethra.

Urethral prolapse has been reported mostly in prepubertal girls and post-menopausal women [1]. Prepubertal girls are usually asymptomatic, while post-menopausal women are often symptomatic [2]. Common symptoms include vaginal bleeding and irreducible painful round doughnut-shaped. Treatment of urethral prolapse should begin with medical therapy in most patients [3]. However, in patients who are symptomatic with severe pain, visible thrombosis, necrosis of the tissue, bleeding, or distress, surgical excision may be a first-line option given the effective time required for estrogen, as well as the low likelihood of successful resolution. Especially in cases of clearly thrombosed or strangulated prolapse, surgical excision should be the first-line therapy [4]. We here report a case of urethral prolapse in a girl, mimicking a uterine prolapse, successfully managed by primarily circumferential excision of the prolapsed urethral mucosa. This case report is reported in line with the SCARE 2020 criteria [5].

## 2 Case presentation

This is a case of an 8-year-old girl, who presented with 2 days history of painful genital hemorrhage and dysuria. There was no history of chronic cough or constipation.

**Fig. 1:**
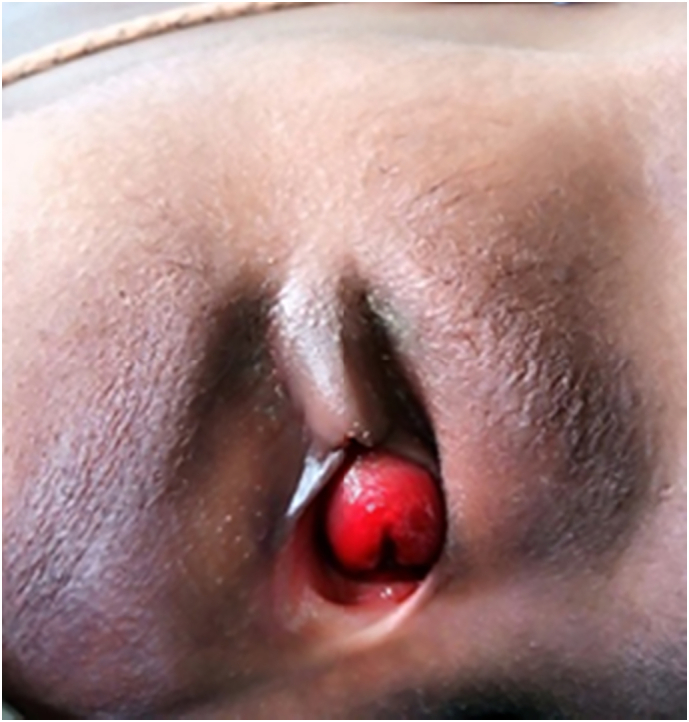
Photography of urethral prolapse on admission.

On examination, we found a bleeding and tender round doughnut-shaped mass (2 × 2 cm) protruding from the vagina, resembling a uterine cervix (Fig. 1).

A pelvic ultrasound scan was done and revealed a normal uterus at its usual location.

**Fig. 2:**
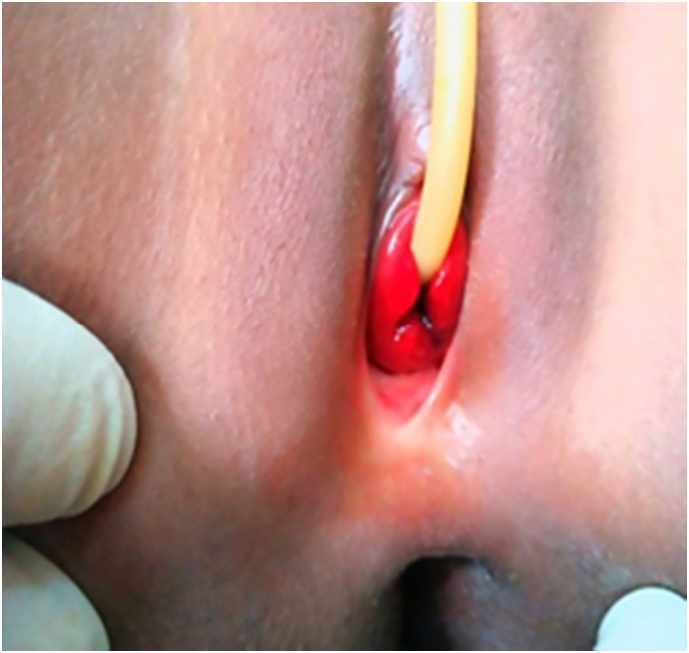
Photography of urethral prolapse after urinary catheter insertion.

A diagnosis of urethral prolapse was made, and the patient was taken to the theatre after her caretaker (her mother) was counseled about the diagnosis, the option management, and eventual complications. We got her informed consent. A preoperative assessment was made. Under general anesthesia, a urinary catheter was inserted into the bladder (Fig. 2).

**Fig. 3:**
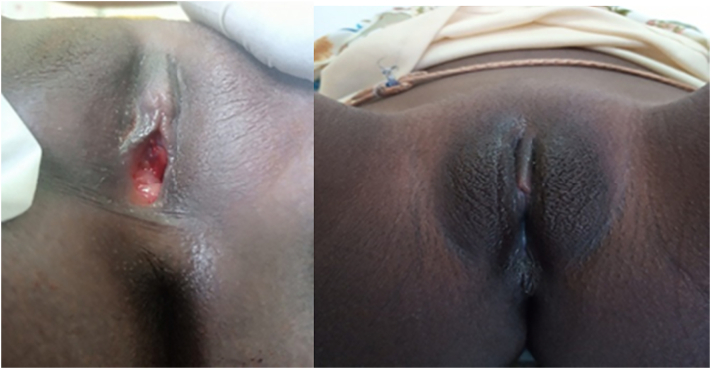
Photography of the vulvar two weeks post-management of the urethral prolapse.

Patient in lithotomy position, a circumferential excision of the prolapsed urethral mucosa was made followed by an interrupted hemostatic suturing using vicryl 4/0. There was no complication, and the blood loss was estimated to be less than 50 ml. The procedure lasted 12 min. The catheter was left in situ for 3 days before being discharged. After then she started applying Premarin cream, a conjugated estrogen cream, twice per day for 2 weeks. She had tender breast bulking and vulvar hyperpigmentation (Figs. 3) secondary to estrogen use.

Three months later she was reviewed and was found asymptomatic.

## 3 Discussion

The etiology of urethral prolapse is not yet well known and many theories are trying to explain its occurrence [6].

A congenital abnormalities theory has been made such abnormal urethral anatomy resulting in a weak attachment between the inner and outer smooth muscle layers of the urethra, a neuromuscular abnormality, and an elastic tissue deficiency [3]. Acquired risk factors include conditions resulting in increased intra-abdominal pressure such as a chronic cough, asthma, obesity, constipation; malnutrition, diarrhea, and urinary tract infection [1], [3], [6].

We did not notice such conditions in our patient. As most cases occur in post-menopausal women and prepubertal girls, it is thought an under-laying low level of estrogen has a role [1], [7].

The clinical diagnosis is not obvious, a high rate of misdiagnosis is observed [3].

Unlike in postmenopausal women, prepubertal girls are asymptomatic and the urethral prolapse is accidentally discovered, as a round, soft, and plain red-colored mass, donut-shaped from the vagina measuring 0.5 to several centimeters in diameter protruding from the distal urethra [3].

Genital hemorrhage is usually not profuse and urinary symptoms such as dysuria, urinary retention can be observed [3].

If there persist some doubts to make the diagnosis, a urinary catheter can be inserted into the bladder through the round mass to confirm that the meatus lay at the prolapsed mucosa.

Treatment of urethral prolapse should begin with medical therapy in most patients [3]. However, Sheng-de Wu et al. in their article on 16-year experience of urethral prolapse management, among 89 cases managed with topical estrogen, 75 cases were recurrence (84% of conservative management failure) ending up in secondary successful surgical management option. This high failure rate was due to severe symptoms of urethral prolapse [3]. This is why it has been recommended primary surgical management be a first-line option in conditions of bigger size, severe genital hemorrhage, and the prolapse with an appearance suggestive of vascular compromise [1].

We also thought applying conjugated estrogen cream could fasten wound healing and prevent recurrence in this young girl supposed with low estrogen levels: estrogens act on the cutaneous wound healing response by modulating the inflammatory response, cytokine expression, and matrix deposition [8]. In addition, estrogens accelerate re-epithelialization, stimulate angiogenesis and wound contraction, and regulate proteolysis [8], [9]. Vaginal estrogen administration is expected to improve healing and long-term maintenance of connective tissue integrity of the pelvic floor [9], [10] and therefore reduce the risk of recurrence.

Our young girl had a 2 cm urethral prolapse mimicking a uterus prolapse. We estimate a surgical excision was the better management option given the size of the prolapsed mucosa and the effectiveness of this option.

## 4 Conclusion

Surgical excision may be a first-line option given the effectiveness of the long-time required for estrogen, as well as the low likelihood of successful resolution, especially in case of thrombosed or strangulated prolapse, severe prolapse mimicking a uterus prolapse, and a high likelihood of recurrence and loss to follow up.

## Consent for publication

Written informed consent was obtained from the caretaker of the patient for publication and accompanying images.

## Funding

This research did not receive any specific grant from funding agencies in the public, commercial, or not-for-profit sectors.

## Ethics approval

Not applicable

## Research registration

Not applicable.

## Guarantor

John Kasereka Muteke.

## Provenance and peer review

Not commissioned, externally peer-reviewed.

## CRediT authorship contribution statement

JKM managed the patient, AA and JKM participated in the follow-up; JKM and FKS wrote the first draft. All co-authors read and approved the final manuscript.

## Declaration of competing interest

The authors declare that there is no conflict of interest in this article.

## References

[1] Holbrook C., Misra D. (2012). Surgical management of urethral prolapse in girls: 13 years' experience. BJU Int..

[2] Hu T.-C., Ker C.-R., Long C.-Y. (2020). Urine retention caused by urethral prolapse mimicking uterine prolapse. Taiwan. J. Obstet. Gynecol..

[3] Wei Y. (2017). Diagnosis and treatment of urethral prolapse in children: 16 years' experience with 89 Chinese girls. Arab J. Urol..

[4] Hall M.E., Oyesanya T., Cameron A.P. (2017). Results of surgical excision of urethral prolapse in symptomatic patients. Neurourol. Urodyn..

[5] Agha R.A. (2020). The SCARE 2020 guideline: updating consensus surgical CAse REport (SCARE) guidelines. Int. J. Surg..

[6] Jessop M.L., Zaslau S., Al-Omar O. (2016). A case of strangulated urethral prolapse in a premenopausal adult female. Case Rep. Urol..

[7] Meutia A.P., Yonathan K., Widia F. (2021). Giant urethral caruncle resembling urethral prolapse causing outflow obstruction. Urol. Case Rep..

[8] Krause M. (2009). Local effects of vaginally administered estrogen therapy: a review. J. Pelvic Med. Surg..

[9] Ashcroft G.S., Ashworth J.J. (2003). Potential role of estrogens in wound healing. Am. J. Clin. Dermatol..

[10] Mukai K. (2016). Evaluation of effects of topical estradiol benzoate application on cutaneous wound healing in ovariectomized female mice. PLoS One.

